# Cancer Stem Cell Immunology: Key to Understanding Tumorigenesis and Tumor Immune Escape?

**DOI:** 10.3389/fimmu.2014.00360

**Published:** 2014-07-29

**Authors:** Valentin S. Bruttel, Jörg Wischhusen

**Affiliations:** ^1^Section for Experimental Tumor Immunology, Department of Obstetrics and Gynecology, School of Medicine, University of Würzburg, Würzburg, Germany

**Keywords:** tumor-propagating cells, tumor immunology, tumor immunosurveillance, tumor dormancy, latency, tumor immune escape, cancer stem cells, cancer stem cell immunology

## Abstract

Cancer stem cell (CSC) biology and tumor immunology have shaped our understanding of tumorigenesis. However, we still do not fully understand why tumors can be contained but not eliminated by the immune system and whether rare CSCs are required for tumor propagation.

Long latency or recurrence periods have been described for most tumors. Conceptually, this requires a subset of malignant cells which is capable of initiating tumors, but is neither eliminated by immune cells nor able to grow straight into overt tumors. These criteria would be fulfilled by CSCs. Stem cells are pluripotent, immune-privileged, and long-living, but depend on specialized niches. Thus, latent tumors may be maintained by a niche-constrained reservoir of long-living CSCs that are exempt from immunosurveillance while niche-independent and more immunogenic daughter cells are constantly eliminated.

The small subpopulation of CSCs is often held responsible for tumor initiation, metastasis, and recurrence. Experimentally, this hypothesis was supported by the observation that only this subset can propagate tumors in non-obese diabetic/scid mice, which lack T and B cells. Yet, the concept was challenged when an unexpectedly large proportion of melanoma cells were found to be capable of seeding complex tumors in mice which further lack NK cells. Moreover, the link between stem cell-like properties and tumorigenicity was not sustained in these highly immunodeficient animals. In humans, however, tumor-propagating cells must also escape from immune-mediated destruction. The ability to persist and to initiate neoplastic growth in the presence of immunosurveillance – which would be lost in a maximally immunodeficient animal model – could hence be a decisive criterion for CSCs.

Consequently, integrating scientific insight from stem cell biology and tumor immunology to build a new concept of “CSC immunology” may help to reconcile the outlined contradictions and to improve our understanding of tumorigenesis.

## Introduction to Tumor Immunosurveillance

Cancer is caused by accumulating genetic alterations in a cell. These lead to activation or multiplication of proteins promoting cell growth and survival whereas proteins promoting cell cycle arrest or cell death are lost or inactivated. Every day no less than one million molecular lesions are thought to occur in the DNA of a single cell (1). While most of these are instantly repaired, the high number implies that very effective control mechanisms must exist to prevent the formation of tumors by mutant cells. This includes both cell-intrinsic mechanisms, such as DNA repair enzymes and tumor suppressor genes [comprehensively reviewed by Hanahan and Weinberg (2)], and the cell-extrinsic control function of the immune system, as first postulated by Paul Ehrlich more than a century ago and systematically introduced by Burnet in 1953 (3). Tumor immunosurveillance relies on traits of malignant transformation that can be recognized by innate and adaptive immune cells. As oncogenic stress induces the upregulation of ligands for activating NK cell receptors (4) and other immune stimulatory surface molecules, tumor cells can be recognized and rejected by NK cells. This innate immune response can further activate a targeted adaptive immune response against antigens specifically expressed by lysed tumor cells and thereby lead to T cell dependent tumor control. Such immunogenic antigens are either highly overexpressed in tumor cells or even tumor specific (when caused by non-silent mutations that have accumulated during malignant transformation) (5). Whole genome sequencing data from lung cancer (6, 7), melanoma, and lymphoblastoid carcinoma (8) genomes indicate that tumors can harbor up to 50,000 somatic mutations of which few hundreds affect protein-coding sequences. It is estimated that this will result in about 7–10 new and unique MHC-binding peptides per HLA allele (9).

Accordingly, innate immunity may constitute a first line of defense, but long-term control is thought to depend on the subsequent adaptive immune responses and on memory cells, which also provide us with life-long protection against pathogens. The detailed roles of different immune cells in tumor immunosurveillance have been comprehensively reviewed recently (10, 11).

Key molecules for tumor immunosurveillance as identified in knockout mouse models comprise interferon-γ (IFNγ) (12), IL-12 (13) perforin (14, 15), TRAIL (tumor necrosis factor related apoptosis-inducing ligand) (16–19), and its corresponding apoptosis-inducing receptors DR4 and DR5 (20), the recombination activating genes RAG1 (21), and RAG2 (12), which are required for T cell development, the T cell receptor (22), and the activating NK cell receptor NKG2D (23). The loss of any of these molecules results in more frequent or faster spontaneous or carcinogen-induced tumorigenesis. The ability to evade immune destruction was thus recognized as an additional hallmark of cancer (24).

## Tumor Latency and Immune Escape

Late recurrence indicates that tumor outgrowth can sometimes be constrained over decades. Koebel et al. (13) elegantly demonstrated that the immune system can control tumor outgrowth without actually eradicating every malignant cell. By treating immunocompetent mice with low doses of the carcinogenic compound methylcholanthrene, they induced stable masses containing only very few malignantly transformed cells. However, when CD4^+^ or CD8^+^ T cells were depleted even as late as 200 days after chemical carcinogenesis, tumors started to grow. This indicates that the immune system can control tumor growth and keep pre-malignant lesions in a dormant state rather than completely eliminating every transformed cell.

Correspondingly, immunocompromised humans such as recipients of organ transplants (25–28) or AIDS patients (29–31) have a significantly increased risk of cancer development, which may be partially attributed to the loss of immunological control over preexistent malignantly transformed cells. Advanced age remains the most important risk factor for tumor development [recently reviewed by de Magalhaes (32)], which may be due to a decline of immunosurveillance. While the stochastic increase in the number of genetic alterations over time and the deterioration of cellular repair mechanisms certainly contribute to this well-known phenomenon, age-dependent defects in the immune system (also termed “immunosenescence”) (33–35) are also likely to facilitate tumor outgrowth. This is corroborated by the finding that adoptively transferred spleen cells from young immunized mice eliminate even large tumors whereas immune cells from old mice are not protective (36). Further factors which may reduce the efficiency of anti-tumor immune responses include non-recognition of tumor antigens due to pre-established tolerance (37), evolution of poorly immunogenic subsets of tumor cells and immunosuppression via accessory cells in the tumor microenvironment. However, tumor-initiating cells need to escape from immunosurveillance even before a proper tumor microenvironment has been established.

On cellular level, continuous genetic transformation can randomly generate less-immunogenic malignant subclones. Lower levels of activatory and/or higher levels of inhibitory NK cell receptor ligands (38, 39) may enable these cells to survive in the presence of the immune system. Also antigens toward which a specific T cell immune response has been established may be lost (40, 41). Furthermore, aggressive tumors are often characterized by low levels of classical HLA class I molecules. Accordingly, those tumor antigens, which are expressed, are poorly presented which limits killing by CD8^+^ cytotoxic T lymphocytes (CTLs) (42). Nevertheless, a complete loss of ”self-MHC,” which would render cells most susceptible for NK cell killing (11, 43–45) does not seem to be the rule. A further important aspect is the active suppression of NK and T cells in the tumor microenvironment where immunosuppressive cytokines or hormones are abundant. Cell surface expression and/or secretion of immune-inhibitory non-classical HLA class I molecules such as HLA-G and HLA-E also contributes to local immune paralysis (42).

Physiologically, many of these factors are required for feto-maternal immune tolerance. As immune-mediated destruction of semi-allogeneic embryonic cells would preclude a successful pregnancy, tolerance toward cells expressing “non-self” antigens is essential for reproduction. This imposes certain limits to the stringent elimination of altered cells. Unfortunately, the same tolerance inducing strategies may also be used by cancer cells to overcome immunosurveillance. Nevertheless, mechanisms to prevent tumor growth still fulfill their evolutionary purpose by largely protecting individuals from cancer until reproductive age or, ideally, until their children (or even grandchildren) have become adults.

## Introduction to Cancer Stem Cells

**Figure 1:**
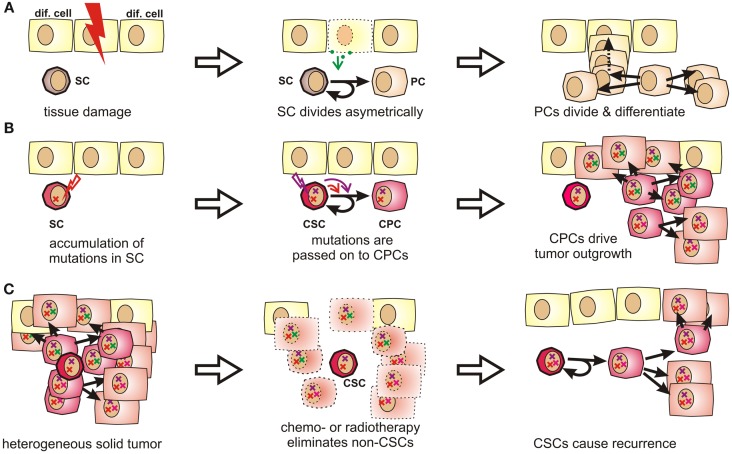
**Stem cells and progenitor cells in tissue homeostasis, tumorigenesis, and tumor recurrence**. **(A)** During organogenesis or in response to tissue damage, resting stem cells (SCs) are induced to undergo asymmetric divisions that produce further differentiated progenitor cells (PCs). Progenitor cells can quickly regenerate tissue by first undergoing a limited number of symmetric cell divisions. The generated daughter cells can then further differentiate to acquire organ-specific functions while generally losing the ability to reenter the cell cycle. **(B)** Stem cells are exceptionally long-lived, pluripotent, and can undergo an unlimited number of cell divisions. Therefore, they may accumulate more mutations than other cells and are thus stochastically more likely to initiate a tumor. These mutations are passed on to progenitor cells, which may drive tumor outgrowth as they cycle much faster by nature. **(C)** Stem cells and CSCs are highly resistant to chemotherapeutic agents and irradiation, which explains why these cells might selectively survive cytoreductive therapy. Therefore, these cells may cause cancer recurrence years or decades later.

Tissue-specific stem cells have been described in many tissues prone to cancer, including breast (46, 47), lung (48), prostate (49–51), intestine (52, 53), and many others. They are exceptionally long-lived and can perpetually self-renew. They further give rise to progenitor cells, which are more restricted in their developmental potential, but capable of undergoing rapid cell divisions. The respective daughter cells then further differentiate into mature tissue-specific cells, which contribute to growth, tissue homeostasis, and wound healing (Figure 1A) [reviewed by Ref. (54, 55)].

This cellular hierarchy within a tissue has been studied most extensively in the hematopoietic system. Here, long term and short term stem cells, multipotent and lineage-committed progenitor cells, precursors, and fully differentiated cells have been well characterized based on their functional properties and on cell surface markers [reviewed by Orkin (56)]. Among these, pluripotent stem cells tend to cycle very slowly while the bulk of blood cells originate from various frequently dividing progenitor cell populations (57, 58). In adult mice, a population of quiescent or dormant hematopoietic stem cells has been described to undergo only about five divisions during the lifespan of a mouse (59).

Because of their longevity, pluripotency, and seemingly unlimited potential to undergo cell divisions, stem cells have long been suspected to be the culprits for tumor formation (60). It is indeed much more likely that a stem cell, which persists for decades in a given tissue, rather than a short-lived differentiated cell, can accumulate numerous mutations as required for malignant transformation (61, 62) (Figures 1B and S1 in Supplementary Material). Moreover, the presence of highly diverse cancer cell populations both in tumors and metastases indicates that their cells of origin are pluripotent. In fact, Kleinsmith and colleagues showed already in the 1960s that some teratocarcinoma cells are capable of seeding highly heterogeneous tumors even if only single cells are transplanted (63). Recently, it was shown that even some blood vessels within glioblastoma are derived from tumor cells (64). This strikingly illustrates the remaining differentiation potential of tumor-initiating cells.

Clinical observations from cancer patients teach us that tumor recurrence may still occur years or decades after a successful-appearing treatment. In this context, a recent sequencing study on acute myeloid leukemia showed that cells collected at the time of the initial diagnosis and during relapse were both closely related to stem cells. Surprisingly, cells collected after relapse had often undergone fewer cell divisions than cells collected at first diagnosis (65). This suggests that the relapsed disease originated from Cancer stem cells (CSCs), which had been largely quiescent during the initial tumor burst, but which survived tumor eradication without losing their malignant potential. Indirectly, this also confirms the existence of a long-lived and multipotent subpopulation of cancer cells, which is highly resistant to radio- and chemotherapy (Figure 1C) and too small to be detected by common screening methods.

Similar to non-malignant stem cells, CSCs are thought to cycle much more slowly than cancer progenitor cells (CPCs). This could explain their resistance toward drugs directed against dividing cells. Additionally, many stem cells can minimize intracellular drug levels via expression of ABC (ATP binding cassette) transporters (66). Accordingly, chemoresistance is expected to be an inherent trait of CSC even before the onset of therapy. Furthermore, stem cells and cancer cells share decreased p53 activity and increased telomerase activity (67).

Still, resistant CSCs are not necessarily the main drivers of rapid tumor outgrowth. While acquisition of specific mutations may alter the replicative behavior of these quiescent and niche-dependent cells (68), homeostatic, and presumably also neoplastic tissue growth depends mainly on niche-independent progenitor cells which divide much more rapidly. In this manuscript, the term CPC is used for the malignant counterparts of such rapidly dividing and lineage-committed cells. As these cells inherit genomic mutations accumulated by stem cells, malignantly transformed stem cells will give rise to malignantly transformed committed progenitor cells, which can then drive tumor growth. Luminal progenitor cells deleted for the tumor suppressor gene Brca1 were thus shown to seed basal-like breast cancer in mice (69, 70). Likewise, prostate cancer can be initiated by basal progenitor cells, which have been virally transfected with oncogenes (71). Obviously, CPCs may not only inherit mutations, but also accumulate “first genetic hits,” which likely contributes to clonal evolution and to the heterogeneity typically found in late stage tumors.

## The Cancer Stem Cell Hypothesis – Ambiguities and Criticism

Cellular differentiation states are difficult to categorize. While omnipotent embryonic stem cells (ESCs) and terminally differentiated cells represent the two ends of the spectrum, numerous gradual transitions exist in between. Defining clear-cut categories is even more difficult in the tumor context where cellular functions and markers may be randomly altered due to mutations. Distinct stem cell markers have been described for CSCs, but these only help to define CSCs within a certain tissue. In an attempt to define the unifying functional characteristics of a CSC, a panel at the American Association of Cancer Research (AACR) meeting suggested in 2006: “A CSC is a cell within a tumor that possesses the capacity to self-renew and to cause the heterogeneous lineages of cancer cells that comprise the tumor” (72). We consider this definition to be insufficient for several reasons:

1) De-differentiation. By now, a considerable body of evidence indicates that lineage-committed differentiated cells and progenitor cells can de-differentiate into multipotent stem cells. While induced pluripotent stem cells [iPS cells as first introduced by Takahashi and Yamanaka (73)] are typically generated by artificially introducing defined transcription factors into differentiated cells, de-differentiation can also occur spontaneously (74) or in response to external stimuli (75, 76). Accordingly, both tissues and tumors may not be organized in a strictly hierarchical manner with few stem cells on the apex, but rather in a more dynamic way with constantly differentiating and de-differentiating cells (77, 78). Likewise, a fairly constant percentage of CSC-like cells can be found to co-exist with basal or luminal progenitor cells in some established tumor cell lines. These populations can be isolated. However, if cultured for a few days, each of them will regenerate the depleted populations and an equilibrium containing almost identical proportions of all three original differentiation states is quickly reestablished (77).
  The most prominent example of physiological transdifferentiation is the so-called epithelial to mesenchymal transition (EMT) (79–81) during which epithelial cells acquire stem cell-like properties. Interestingly, in breast cancer or melanoma EMT can be induced by CD8^+^ T cells (77, 80). Accordingly, there appears to be more plasticity within the cellular hierarchy than previously anticipated, especially in tumors.
2) The frequency of tumor-propagating cells depends on the respective animal model. For melanoma, CSC markers, frequencies, and their role in tumorigenesis were investigated in considerable detail. CD20 (82), CD133 (83), ABCG2 (83), MDR1 (84), or CD271 (85) have all been described as potential markers for melanoma cells with increased tumorigenic potential as assessed in non-obese diabetic (NOD)/severe combined immune-deficient (scid) mice. With about one CSC in 10^6^ melanoma cells, these CSCs constitute only a minute subset of all tumor cells (86). However, in a highly debated (87, 88) article, Quintana et al. (89) reported that about 27% of highly aggressive melanoma cells were capable of inducing tumors in interleukin-2 receptor gamma chain deficient (Il2rg^−/−^) NOD/scid (NSG) mice, which do not only lack T and B, but also NK cells. Thus, the proportion of tumor cells, which can self-renew and seed heterogeneous tumors in highly immunodeficient mice may be much higher than previously anticipated. This raised the question whether the small subpopulation of CSCs is of any clinical relevance (86).
  In a later study, the same authors proposed that most melanoma cells may reversibly express stem cell markers (90). They argue in favor of clonal evolution and negate any kind of hierarchical organization within a tumor cell population, thus refuting the basic idea underlying the CSC hypothesis. Accordingly, the term CSC is avoided by some authors and replaced by more descriptive alternatives such as cancer-initiating cell, tumor-propagating cell or metastasis-initiating cell. In our opinion, however, this approach has not helped to simplify matters, especially since suitable markers and physiologically correlating cell populations are ill-defined.

We believe that the ability of differentiated cancer cells to de-differentiate into CSCs may partly reconcile these conflicting findings. Moreover, it should be noted that these data were generated in mice with very different immunological properties. Thus, a unique and functionally most relevant criterion shared by stem cells and CSCs, namely their lack of immunogenicity, should be considered.

## Immunological Properties of CSCs

Unfortunately, our knowledge about specific immunological properties of distinct CSC populations is still limited. It is, however, clear that high levels of anti-apoptotic proteins like bcl-2, bcl-xL, or survivin do not only protect CSCs against chemotherapeutic drugs (91), but also increase resistance toward apoptosis-inducing immune effectors like T or NK cells. In this context, the PI3K/Akt pathway seems to be of particular relevance: it is not only a known mediator of chemoresistance and CSC renewal (92), but was also found to be involved in tumor immune escape (93). Likewise, the oncogenic growth factor receptor HER2/neu (which activates PI3K/Akt signaling) does not only deliver pro-survival signals (94). HER2 does also interfere with antigen processing and presentation (95, 96) and it was reported as crucial for the maintenance of CSC in luminal breast cancer (97). Thus, mechanisms which were mainly thought to mediate chemoresistance in CSC may also confer cross protection against immune-mediated tumor eradication.

In the aggressive melanoma cell line A375, Schatton and Frank also found the immunogenic tumor-associated antigen MART-1 (melanoma antigen recognized by T cells) to be only expressed on differentiated melanoma cells, but not on malignant melanoma initiating cells (MMICs). MART-1-specific T cells thus cannot eliminate MMICs (98). Reim et al. have further shown that CD44^high^/CD24^low^ breast CSCs selectively escape from NK cell mediated killing and trastuzumab-dependent ADCC (99). While these findings explain the difficulties in targeting CSCs with immune cells, stem cell-like cells are not only poor targets for immunosurveillance. They also actively suppress immune responses (100), which may be crucial during tumorigenesis (101). Breast CSCs (102) and glioblastoma stem cells (103) secrete more TGF-β as compared to normal tumor cells. Colon CSCs are further known to secrete Interleukin 4 (104), which promotes drug resistance (104) and inhibits anti-tumor immune responses (105). CSCs also express CD200 (106), a molecule that inhibits myeloid cells and could therefore play a major role in tumor immune escape (107, 108). Immunological properties of CSCs in solid tumors have also been recently reviewed by Maccalli et al. (109).

More is known about molecular immunological properties of physiological stem cells. ESCs express little classical antigen-presenting MHC class Ia molecules and no MHC class II molecules and will thus be poor antigen presenters for T cells. Additionally, they express only low levels of ligands for the activatory NK cell receptor NKp44 and no ligands for NKp30, NKp46, and CD16 (110). Among inhibitory signals, HLA-G (111) is highly expressed by both embryonic (112) and mesenchymal stem cells (MSCs) (113). Thus, studies describing higher MHC class I expression on stem cells may have failed to distinguish between immunogenic MHC class Ia (HLA-A, -B, -C) and immunosuppressive MHC class Ib (HLA-E, -F, -G) molecules. MHC class II or costimulatory molecules such as CD40, B7-1 (CD80), or B7-2 (CD86), which can also support T cell interactions with non-lymphoid cells (114) have not been found on MSCs (115–117).

T cell proliferation, macrophage activation, and Th1 responses can further be suppressed by prostaglandin E2 (PGE2), which is secreted by MSCs (118, 119). After exposure to IFN-γ, MSCs also upregulate indoleamine 2,3-dioxygenase (IDO), which metabolizes tryptophan into immunosuppressive kynurenines. The concomitant tryptophan depletion also has a profound inhibiting effect on T cells (120). Via secretion of hepatocyte growth factor (121), MSCs can further induce tolerogenic dendritic cells and regulatory T cells (122). Consequently, MSCs show beneficial effects in autoimmune disease models (123–125).

**Figure 2:**
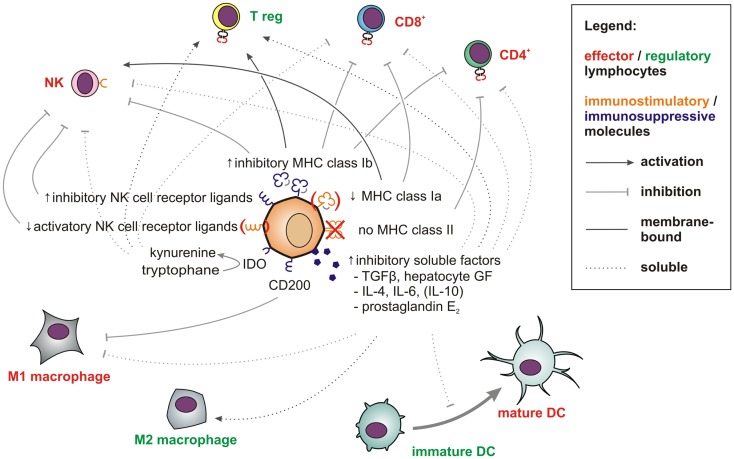
**Factors and pathways contributing to the low immunogenicity of stem cells and CSCs**. Membrane-bound (solid line) and soluble factors (dotted lines) implicated in immunotolerance toward stem cells and CSCs are schematically depicted. These include antigen-presenting and immunostimulatory molecules (orange) expressed at low levels and abundantly expressed immunosuppressive factors (blue) (see main text for references). Immune effector cells impaired by these signals are shown in red whereas tolerogenic immune cells, which may be stimulated are displayed in green.

To summarize, stem cells and CSCs both express numerous and diverse membrane-bound and soluble factors (Figure 2), which enable these cells to efficiently modulate immune responses and which protect them against immune-mediated destruction in a way that is unrivaled by further or fully differentiated cells. Strikingly, mouse ESCs and human MSCs have even been shown to survive for weeks and to engraft in immunocompetent rats (126) and sheep (127, 128). The immunomodulatory potential of MSCs is now exploited clinically to counteract graft-versus-host disease (129) and to promote allograft acceptance upon solid organ transplantation (130).

As the immune system is incapable of eliminating allogeneic or even xenogenic stem cells, the comparatively few mutations acquired by CSC are unlikely to cause immune-mediated rejection. Thus, stem cells likely represent a unique compartment in which oncogenic mutations can accumulate without being detected by the immune system.

## Immune Privilege – The Missing CSC Criterion?

**Figure 3:**
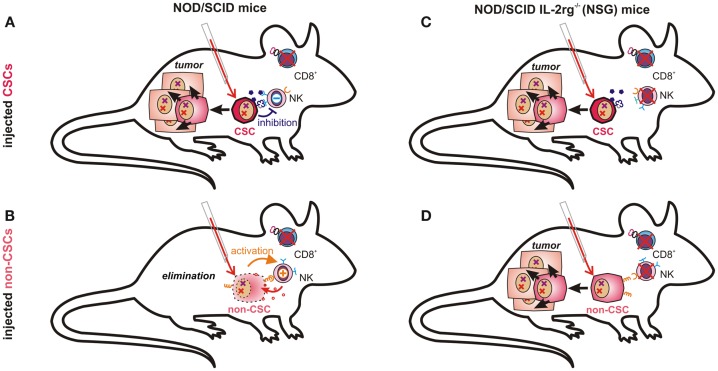
**Tumor-propagating capacity depends on immunological properties of injected cancer cells and on the respective mouse model**. NK cells in NOD/scid mice are likely incapable of eliminating CSCs due to their low immunogenicity **(A)**. More differentiated cancer cells expressing ligands for activating NK cell receptors and fewer immunosuppressive molecules may, however, be recognized and eliminated by host NK cells **(B)**. In NOD/scid IL2rg^−/−^ (NSG) mice, which also lack NK cells, both poorly immunogenic CSCs **(C)** and further differentiated cancer cells **(D)** can seed tumors, as malignant progenitor cells may also possess enormous proliferative capacity.

The question whether or not all cancer cells are equally capable of seeding tumors obviously depends also on their ability to escape from destruction by the innate or adaptive immune system. This essential difference, however, may not be observable in a mouse model lacking functional T, B, and NK cells, such as the NSG (131) mice used by Quintana et al. (89). The conflicting data on the frequency of melanoma initiating cells can thus easily be explained by accepting that CSCs are much less sensitive toward tumor immunosurveillance than more differentiated cells. While NK cells in NOD/scid mice may clear more differentiated cancer cells (Figure 3B), they cannot eliminate immunosubversive human CSCs (Figure 3A). Thus, non-CSC-like cancer cells may replicate endlessly *in vitro* whereas only CSCs will seed tumors in these mice. NK cell-deficient NSG mice can, in contrast, neither eliminate CSCs (Figure 3C) nor more differentiated cancer cells, which would then also become capable of seeding tumors (Figure 3D). Their recently discovered ability to de-differentiate may then further facilitate tumor propagation.

A maximally immune-deficient mouse may therefore demonstrate the malignant potential of differentiated cancer cells in the complete absence of immunosurveillance, an aspect that may have been underestimated in the original CSC theory. We, however, wonder how relevant NSG mice can be for understanding tumor initiation (and thus tumor-initiating cells) in patients. While stem cell experts tend to favor the most completely immunodeficient animal model available, the most relevant subject of translational cancer research is the immune-competent human subject afflicted by a malignant disease. The ability to propagate tumors should therefore best be tested in models possessing a functional immune system. Furthermore, as implied by studies performed in NOD/scid mice (132–134), the ability to continuously seed tumors in presence of (residual) immunosurveillance may be a most relevant functional criterion for CSCs.

Consequently, tumor initiation may better be analyzed in congenic or syngenic animals rather than in immunodeficient xenograft models. Limitations arise from the fact that all cells contained in transplantable syngenic tumor cell lines have evidently undergone immunoediting *in vivo* before the cell line could be derived. Accordingly, the proportion of immune-refractory, tumor-seeding cells may be quite variable: with B cell lymphoma cells, for example, inoculation with 10 unsorted cells was sufficient to induce lethal lymphomas within a few weeks, irrespective of expression of the stem cell marker CD93 (135). In the 4T1 mammary carcinoma cell line, however, exclusion of the stem cells (Hoechst 33342 side population) by cell sorting greatly reduced both tumor take and tumor load, and most animals injected with 8 × 10^3^ non-CSC remained tumor-free (136). Thus, the frequency of CSCs can differ widely depending on the respective tumor.

## Tumor Immunoediting – The Model and Unresolved Questions

The immune privilege of CSCs may not only be relevant for the quantification of tumor-propagating cells, but could also help to elucidate ambiguities in tumor initiation and immune escape. The complex interactions between tumors and the immune system have been described by a model which differentiates between three phases of tumor immunoediting: elimination, equilibrium, and escape (137). There is now solid scientific evidence for the existence of all three phases and this model nicely correlates with clinical observations (10).

During the elimination phase, tumors may be successfully detected and destroyed by the innate and adaptive immune system, which results in the reestablishment of healthy tissue. If, however, some tumor cells escape from elimination, a dynamic equilibrium between tumor growth and immunological elimination of malignant cells was proposed to emerge. While tumor outgrowth is still constrained, elimination of tumor cells remains incomplete, which allows for mutational diversification and Darwinian selection to occur. This latency phase may persist for decades and may be functionally similar to tumor dormancy, the time between an initially successful anti-tumor therapy and tumor recurrence, which can also span decades (138). When ultimately one clone arises that is capable of escaping from a functional immune system, it may form a clinically overt tumor and initiate the escape phase.

However, for the equilibrium to be reached, this model requires “not yet immunoedited” cancer cells to survive the early elimination phase. Furthermore, it is not clear why cells, which can be immunologically constrained in equilibrium for decades, are not simply cleared by an adaptive immune response. Normally, expanded and activated CTLs efficiently eliminate cells expressing their cognate antigen whereas cells that have lost all immunologically relevant antigens could directly grow into overt tumors.

**Figure 4:**
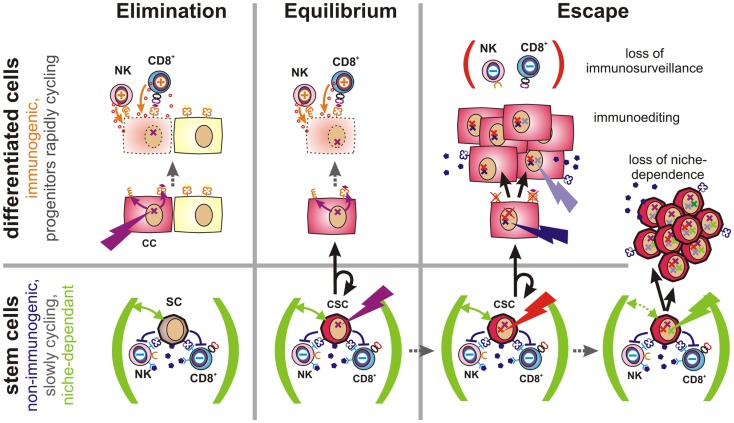
**Proposed role of CSCs in tumor immune escape**. Elimination (Left): malignant transformation requires oncogenic mutations (flash) to accumulate within an individual cell. If such mutations occur in a differentiated cell, this cell will upregulate activatory NK cell receptor ligands and present tumor-specific peptides via MHC class Ia molecules (both orange). Thus, these cells can be detected and eliminated by the immune system, leading to restoration of normal tissue. Equilibrium (Center): stem cells (SC) are long-lived and express a multitude of immunosuppressive factors (dark purple). Hence, they may accumulate oncogenic mutations without being cleared by the immune system. However, CSCs are initially confined to stem cell niches (green) and limited to asymmetric divisions. More differentiated daughter cells inherit all malignant mutations, but are more immunogenic and could thus be eliminated by the adaptive immune system. Thus, a robust equilibrium may emerge. Escape (Right): mechanisms contributing to tumor immune escape include defects in tumor immunosurveillance, immunoediting, or the expansion of CSCs. The immune system may lose its ability to constrain tumors due to aging, immunosuppressive therapies, diseases or other factors. Immunoediting describes the evolutionary adaptation of individual tumor subclones to the selection pressure exerted by the immune system. It will ultimately lead to expansion of less-immunogenic or more immunosuppressive subclones. Non-immunogenic CSCs may further acquire the ability to expand independently of their niches, which may lead to the outgrowth of poorly differentiated tumors.

Based on these considerations, latency requires the existence of a poorly immunogenic sub-pool of cancer cells, which escape from targeted immune responses, which can give rise to new cancer cells, but which are initially incapable of growing into massive tumors. Instead, these cells apparently persist for months, years, or even decades within tissues as dormant tumor cells. In our opinion, multipotent stem cells and CSCs, which are not only long-lived, but also exceptionally well protected from immune-mediated destruction, and largely confined to stem cell niches (68, 139), would meet all these criteria. By considering the immunological properties of CSCs, a new refined concept of tumor immunoediting can thus be proposed (Figure 4 and more detailed in Figure S1 in Supplementary Material).

## Implementing CSCs in the Concept of Tumor Immunoediting

When oncogenic mutations occur in fully or partially differentiated cells, activatory NK cell receptor ligands are induced (4) and tumor antigens are presented via MHC class Ia. Thus, altered cells may be readily detected and eliminated by the immune system. Failure may be due to de-differentiation of mutated cells.

If, instead, an oncogenic mutation occurs in a poorly immunogenic stem cell, escape from immunosurveillance is much more likely. However, their dependence on stem cell niches may prevent mutated stem cells from growing into overt tumors. Upon asymmetric division, a more differentiated and more immunogenic daughter cell will be generated, which would then be subjected to immunosurveillance. As the other daughter cell will maintain stem cell characteristics, a robust equilibrium would emerge. Recent findings indicate that AML finally arises from such pre-malignant stem cells (140).

During this latency period, stochastic genetic and epigenetic changes may accumulate within the reservoir of non-immunogenic stem cells. Mutations in coding regions may generate new immunogenic peptides that would be presented on MHC molecules of malignant daughter cells. Such neoantigens may support immunosurveillance by providing further tumor antigens and, ideally, enabling tumor clearance. However, if CSCs are not eliminated by the immune system, the low frequency of niche-confined, mutated stem cells, and their low rate of cell divisions would still prevent the formation of a clinically relevant tumor as long as their daughter cells are subjected to immunosurveillance. Accordingly, the immune system could constrain a sub-clinical malignant disease in life-long equilibrium.

However, once tumors become clinically apparent immunosurveillance has failed. This can be due to a weakening of the immune system caused by aging, to therapeutic immunosuppression or to disease. In this case, also moderately immunogenic tumor cells may propagate. Alternatively, cancer cells may actively attenuate immunosurveillance (immunosubversion) by secretion or expression of immunosuppressive factors (11) or by recruitment of accessory cells, which locally suppress the immune system (141, 142). Furthermore, genetic alterations may result in reduced expression of antigens that are targeted by the immune system (immunoselection). Other factors allowing for the rapid growth of more differentiated cancer cells include the loss of the antigen processing and presentation machinery (143) or the loss or shedding of ligands for activatory NK cell receptors (144). Importantly, non-immunogenic long-lived CSCs provide an evolutionary playground for mutations to accumulate until an immune escape phenotype has emerged.

Expansion of immune-evasive CSCs represents a further possibility for immune escape. This mechanism may predominate in poorly differentiated tumors, which tend to be more aggressive and more difficult to treat (145). Alterations in cell signaling pathways are known to enable CSCs to grow outside of their niches (68). Interestingly, signals that promote stem cell self-renewal and expansion (146) are also frequently activated in cancer as evidenced by alterations in the notch, wnt, and sonic hedgehog pathways (147–149).

Oncogenic mutations may also increase the frequency of cell divisions in otherwise fairly quiescent stem cells (150) or inhibit differentiation in the respective daughter cells (151, 152). Furthermore, CSC proliferation may be promoted by conditions like chronic inflammation, which triggers physiological stem cell division. As these mechanisms are not mutually exclusive, increased cell division in CSCs will also increase the risk for emergence of less-immunogenic and more aggressive subclones.

## Implications of “Cancer Stem Cell Immunology”

Stem cell research has helped to understand tumor initiation, heterogeneity, and drug resistance, while immunological knowledge has provided a framework for apprehending mechanisms that naturally limit tumor growth. Consolidating insights from these two different scientific fields may further help to explain phenomena which are still poorly understood.

As discussed in this opinion article, we propose that pluripotency, immortality, and an apparently unlimited potential to undergo cell divisions may be insufficient criteria for distinguishing CSCs from other cancer cells. In the tumor context, partly differentiated cells may also acquire these properties through mutations or de-differentiation processes. However, the unique immunological properties of stem cells and CSCs may help to further discriminate these cells from the tumor bulk (Table 1). Experimental evidence supporting this novel perspective may be drawn from model-dependent differences regarding the respective frequencies of tumor-initiating cells. This may best be explained by considering the different levels of immunogenicity coexisting within the same tumor. In addition, CSCs excellently complement current models of tumor immune escape. They can be seen as highly resistant and long-lived units of Darwinian selection, which accumulate mutations and undergo immunoediting over decades. Thus, they can either remain dormant or represent an almost invincible backbone of tumorigenesis which links “the three *e*’s of cancer immunoediting”: elimination, equilibrium, and escape, i.e., tumor recurrence.

**Table 1:** **A grossly schematic overview summarizing characteristic properties and functions of stem cells, progenitor cells, and differentiated cells and their malignant counterparts**.

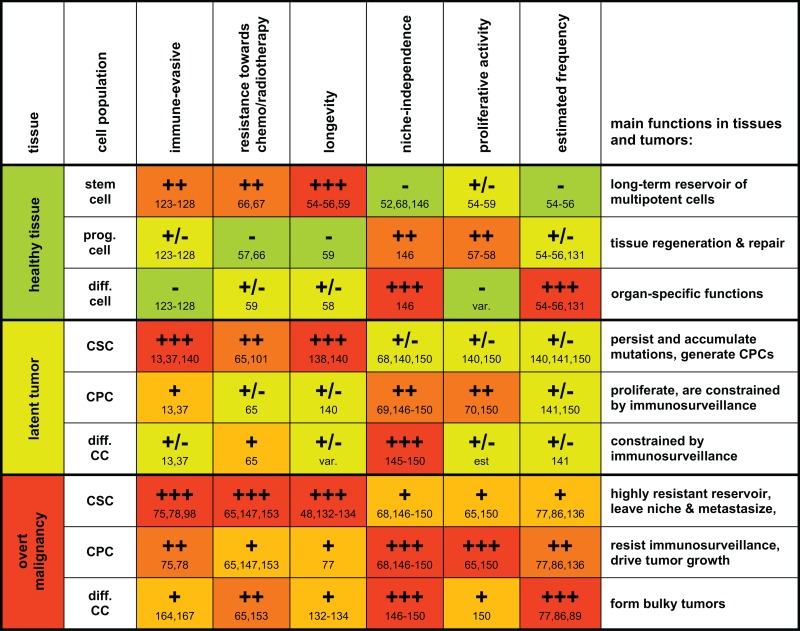

*Properties promoting tumor growth are shaded in red whereas traits that limit the malignant potential are highlighted in green. Though the actual characteristics vary significantly between different tissues and tumors, common tendencies were sought. Some selected references are provided, and more are given in the main body of the manuscript. When no representative reference could be found in the literature, this variability is indicated (var.). Where no data were available, an estimate is given (CSC, cancer stem cell; CPC, cancer progenitor cell; diff. CC, differentiated cancer cell)*.

Yet what are the clinical implications if CSCs are not only highly resistant to chemo- and radiotherapy (153) but also to anti-tumor immune responses? Strategies to specifically target CSCs for elimination (154, 155) or to induce differentiation (156) would obviously be most desirable. However, none of the current approaches shows a high selectivity for CSCs over non-malignant stem cells. Therefore, therapies that efficiently and selectively eliminate CSCs are still far from clinical application. Still, accepting that we are currently unable to eradicate CSCs is far from accepting defeat in the war on cancer. Instead, by accepting these limitations one can rather prioritize strategies, which are more likely to work. These include, on the one hand, the indirect targeting of CSC via the tumor stroma, which represents a niche for CSCs (157–160). On the other hand, containment and control of CSCs to maintain tumors in the equilibrium phase may be a realistic clinical aim. This, however, depends on the incessant functionality of the immune system (161). Beneficial effects of radio- or chemotherapy, which can destroy some tumor cells (but presumably not the CSC) may thus largely depend on immune responses induced by provision of danger signals and by liberation of antigens from damaged tumor cells (37, 162, 163). Strengthening of cancer immunosurveillance by vaccination strategies or by directly activating the immune system (164–166) may support such a response and thus be effective in preventing tumor recurrence over prolonged periods of time (167, 168). Adjuvant immunotherapy might hence be advisable when a successful-appearing treatment has reduced overt cancer to minimal residual disease (which likely represents an immunologically sustained equilibrium). Overaggressive treatment modalities are, however, strongly immunosuppressive and may be detrimental. In fact, a comparatively mild metronomic chemotherapy may often be more effective (169, 170) than application of the maximum tolerated dose, which still fails to eliminate the seed of the evil but enables the outgrowth of more differentiated malignant cells. Assuming that CSCs represent moving targets, which need to be controlled throughout the whole life, we need to learn how the highly dynamic and adaptable forces of our immune system can best be used in the war on cancer. Fortunately, first studies are showing the potential of cancer immunotherapy in clinical reality.

Last but not least, an improved understanding of the mechanisms which enable either tumor control or escape may help to devise suitable therapeutic strategies. We thus hope that the still speculative, but hopefully coherent conceptual framework outlined in this brief article will stimulate further research in “CSC immunology.” In the best case, a mutual exchange of ideas could not only inspire both the CSC and the tumor immunology community, but eventually also lead to some benefit for patients who depend on a wisely chosen therapy.

## Conflict of Interest Statement

The authors declare that the research was conducted in the absence of any commercial or financial relationships that could be construed as a potential conflict of interest.

## Supplementary Material

The Supplementary Material for this article can be found online at http://www.frontiersin.org/Journal/10.3389/fimmu.2014.00360/abstract
