# Supplementary material for: Cancer Stem Cell Immunology: Key to Understanding Tumorigenesis and Tumor Immune Escape?
Source: Front Immunol. 2014 Jul 29;5:360. doi: 10.3389/fimmu.2014.00360 (PMC4114188; doi:10.3389/fimmu.2014.00360)
Supplement: Supplementary file 1 [file Presentation_1.PDF]

Supplementary Figure 1

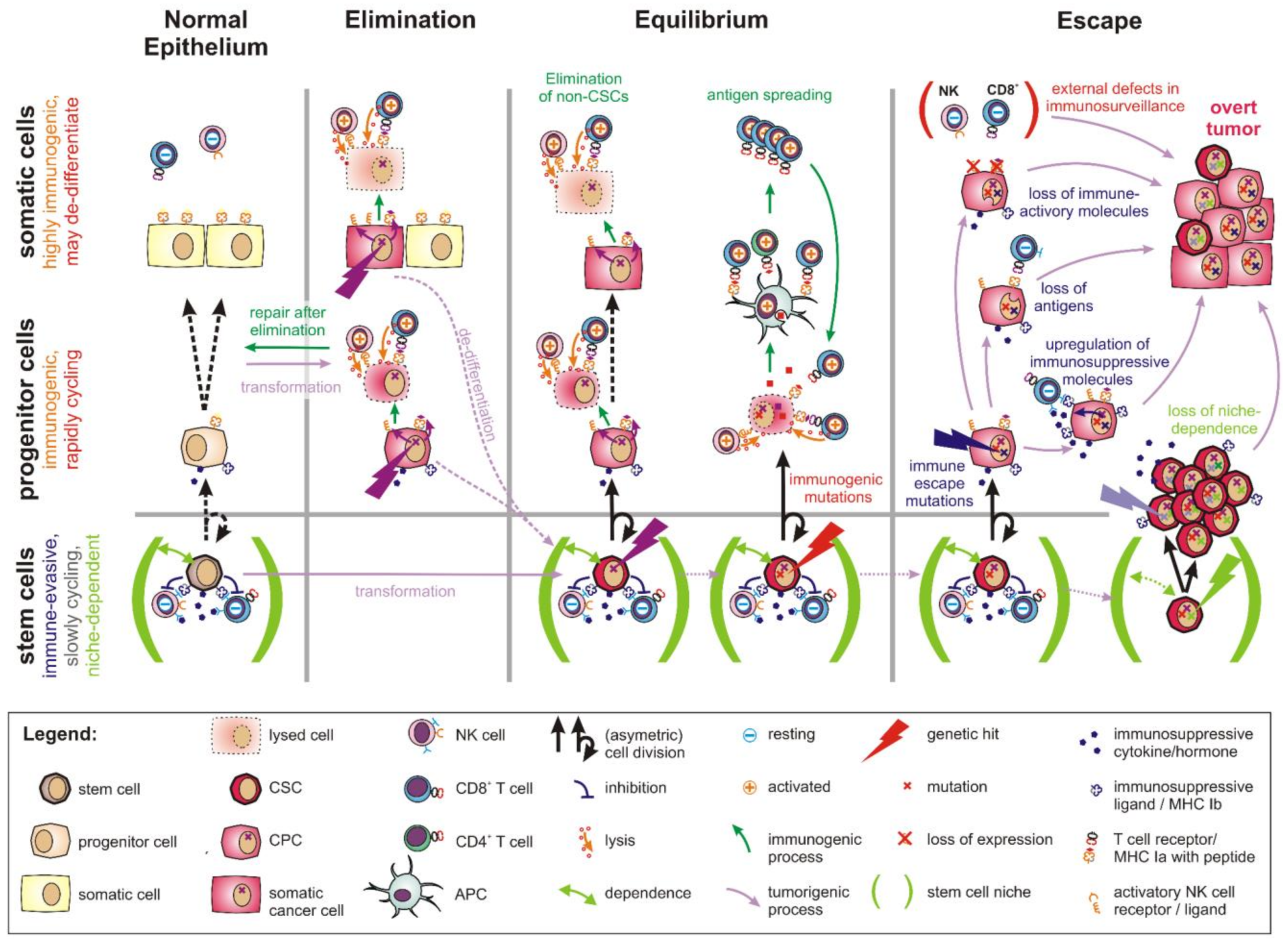

Supplementary Figure 1. Proposed role of stem cells, progenitor cells and somatic cells in tumor immune escape.

From left to right:

Tissue Homeostasis in Normal Epithelium. During organogenesis or in response to tissue damage, rare stem cells are activated to undergo asymmetric divisions that produce more differentiated progenitor cells. Progenitor cells regenerate tissue through symmetric cell divisions. Thus generated daughter cells further differentiate and acquire organ-specific functions while generally losing the ability to reenter the cell cycle. When healthy cells acquire mutations, malignant transformation can occur as a multi-step process which can be divided into different phases:

Elimination. Malignant mutations occurring in somatic cells or progenitor cells are likely to be detected by the immune system due to oncogenic stress-induced ligands for activatory NK cell receptors and tumor antigen presentation via MHC class Ia. These cells are subsequently eliminated, resulting in the restoration of normal epithelium. However, mutated cells may in some cases escape from immunosurveillance by de-differentiating into less immunogenic stem cells.

Equilibrium. If an oncogenic mutation occurs in an immune-evasive stem cell, this cell may not be eliminated by the immune system due to its low expression of immunostimulatory and

high expression of immunosuppressive molecules. However, such premalignant stem cells may not immediately grow into overt tumors as they cycle slowly, depend on stem cell niches and naturally undergo only asymmetric divisions. Their more immunogenic daughter cells, however, may constantly be eliminated by the adaptive immune system. Over decades, such mutated stem cells can accumulate further genetic alterations. Thus, novel tumor-specific antigens may emerge, which can be taken up and presented by APCs to induce activation and expansion of tumor-specific T cells. This would lead to antigen spreading which strengthens tumor immunosurveillance and stabilizes the equilibrium.

Escape. Immune escape mutations occurring in CSCs or premalignant daughter cells may finally lead to tumor outgrowth. Particular risks may be associated with genetic alterations leading to the loss of immune-activatory molecules (like MHC molecules or NK cell receptor ligands), or to loss of immunogenic tumor antigens or to the upregulation of immunosuppressive factors. Furthermore, immunosenescence or therapeutically induced immunosuppression may limit immunosurveillance and thus enable more immunogenic tumors to grow. Alternatively, poorly immunogenic CSCs may lose their niche dependence and expand to form poorly differentiated tumors.
